# Clinical characteristics of 14 cases of severe *Chlamydia psittaci* pneumonia diagnosed by metagenomic next-generation sequencing: A case series

**DOI:** 10.1097/MD.0000000000029238

**Published:** 2022-06-17

**Authors:** Anbing Zhang, Xiuqiong Xia, Xiaoling Yuan, Yanhua Lv, Yuxia Liu, Haiming Niu, Dandan Zhang, Jianping Liang

**Affiliations:** a Department of Respiratory and Critical Care Medicine, Zhongshan People's Hospital, Zhongshan, People's Republic of China; b Department of Intensive Care Unit, Zhongshan People's Hospital, Zhongshan, People's Republic of China.

**Keywords:** *Chlamydia psittaci*, doxycycline, metagenomic next-generation sequencing, quinolone, severe pneumonia

## Abstract

**Introduction:**

The objective of this study was to explore the clinical, laboratory, and imaging features of severe *Chlamydia psittaci* pneumonia in order to improve early diagnosis and treatment success rates.

**Methods:**

We conducted a retrospective record review of 14 cases of severe *Chlamydia psittaci* pneumonia diagnosed by metagenomic next-generation sequencing technology in our hospital. We extracted and analyzed data on the clinical symptoms and signs, contact history, laboratory investigations, chest computed tomography, treatment, and clinical outcomes.

**Results:**

Of the 14 patients, 12 (86%) were male and two (14%) were female, with a mean age of 57 years (SD: 7 years). Eleven patients (79%) had a history of poultry contact. The main clinical manifestations were fever (n = 14, 100%), flu-like symptoms (n = 10, 71%), cough, sputum (n = 9, 64%), and dyspnea (n = 5, 36%). Blood tests revealed marked elevation of neutrophil percentage, C-reactive protein, procalcitonin, brain natriuretic peptide, and creatine kinase levels; slight elevation of aspartate aminotransferase, creatinine, urea, fibrinogen, and D-dimer levels; and decreased albumin, sodium, and calcium levels. Chest computed tomography showed bilateral lesions (n = 7, 50%), middle-lower lobe lesions (n = 10, 71%), lesions in multiple lobes (n = 9, 64%), consolidation shadows (n = 11, 79%), and pleural effusions (n = 11, 79%). The median time from disease onset to hospital admission was 4.5 days (interquartile range: 1–17 days); the mean length of hospital stay was 20.9 ± 8.5 days, and the mean time from admission to diagnosis was 5.1 ± 2.6 days. After diagnosis, patients were either treated with doxycycline alone or doxycycline combined with quinolones. All 14 patients developed respiratory failure and received invasive mechanical ventilation; two (14%) received veno-venous extracorporeal membrane oxygenation, four (29%) received continuous renal replacement therapy, and three (21%) died.

**Discussion and conclusion:**

A poultry contact history and typical flu-like symptoms are early indicators of *Chlamydia psittaci* pneumonia. Substantial elevations in procalcitonin, creatine kinase, and brain natriuretic peptide indicate severe disease. Metagenomic next-generation sequencing is useful for diagnosis. Early empirical antibiotic therapy with quinolones can reduce the mortality in critically ill patients.

## 1 Introduction

*Chlamydia psittaci* is an obligate, intracellular gram-negative bacterium that mainly infects poultry but can also cause zoonotic infections. The inhalation of aerosols contaminated by the excreta of infected birds can cause *Chlamydia psittaci* pneumonia, which can sometimes lead to severe pneumonia and even death.^[1]^ The level of awareness of *C. psittaci* pneumonia among clinicians is generally low, and its clinical presentation is atypical. Consequently, the disease is easily missed and misdiagnosed. If the diagnosis is delayed, patients may develop severe illness. The traditional culture of *C. psittaci* is associated with strict requirements, the sensitivity is low, and most laboratories do not routinely culture for *Chlamydia*. Serological test and polymerase chain reaction testing is available at some medical institutions, but their sensitivity and specificity are generally low.^[2]^ Metagenomic next-generation sequencing (mNGS) has gradually been adopted for the diagnosis of difficult to diagnose, severe pneumonia in recent years.

We retrospectively analyzed the clinical data of 14 patients with severe *C. psittaci* pneumonia diagnosed by mNGS. The purpose of this report is to help clinicians with the early identification of patients with this disease, and to describe our experience with early administration of empirical antibiotic treatment, in order to improve treatment success rates.

## 2 Subjects and methods

### 2.1 Research subjects

Fourteen patients with severe *C. psittaci* pneumonia who were admitted to our hospital from January 2019 to November 2021 and met the inclusion criteria were selected for the review. The inclusion criteria for were as follows:

(1) complied with the 2019 Infectious Diseases Society of America/American Thoracic Society Guidelines for the diagnosis and treatment of adult community-acquired pneumonia (CAP);^[3]^
(2) fulfilled the diagnostic criteria for severe pneumonia;^[3]^
(3) identification of the *C. psittaci* gene fragments through mNGS analysis of the bronchoalveolar lavage fluid (BALF), blood, or sputum, and met the criteria for a positive mNGS result;^[4]^
(4) routine microbiological tests, including blood, sputum, and alveolar lavage fluid culture were all negative; and
(5) respiratory failure, defined as an oxygenation index <400 mm Hg (1 mm Hg = 0.133 kPa).

This study was approved by the Ethics Committee of the Zhongshan People's Hospital (approval no.: K2021-127) and proceeded in accordance with the Declaration of Helsinki. All patients or their family members provided signed informed consent. All data in this study were anonymized before analysis.

### 2.2 mNGS of alveolar lavage fluid

The mNGS utilized for this study was completed in collaboration with the Shenzhen Huada Gene Technology Co. Ltd. (Shenzhen, China). DNA extraction was performed by transferring 3 to 5 mL of alveolar lavage fluid into a centrifuge test tube, placing the test tube at 4°C and centrifuging at a high speed of 4000 rpm for 10 minutes, in accordance with the instructions of the TIANamp Micro DNA Kit (DP316, Tiangen Biotech, Beijing, China). The extracted DNA samples were broken into fragments of approximately 150 bp ultrasonically. An Agilent 2100 Bioanalyzer QC (quality control) library with an insert size of 200 to 300 bp was used; the Qubit dsDNA HS Assay Kit (Thermo Fisher Scientific Inc.) was used to control the DNA library concentration, and cyclization was performed to form a single-chain ring structure. The cyclized library underwent rolling circle replication to generate DNA nanoballs. The prepared DNA nanoballs were loaded into the sequencing chip for sequencing, using a BGISEQ-50/MGISEQ-2000. Low-quality short fragments (length <35 bp) were removed after the sequencing data were derived from the machine to obtain high-quality sequencing data. Burrows-Wheeler Aligner (BWA) (BWA: http://bio-bwa.sourceforge.net/) sequencing analysis software was used to remove data that were matched with the reference human genomic sequence after a comparison with high-quality data. The remaining data were compared with the four microbial databases of bacteria, fungi, viruses, and parasites. After removing low-complexity sequences, the number of sequences that matched certain pathogens was obtained, and the possible pathogens, based on the number of sequences and other clinical tests, were determined.

### 2.3 Data collection

The demographics of patients, such as sex, age, body mass index, smoking history, and poultry contact history, among others, were collected. The clinical manifestations, physical signs, laboratory examinations, and imaging examination results, as well as the patients’ pneumonia severity scores, acute physiology, and chronic health scores, were recorded.

### 2.4 Laboratory and imaging tests

On the day of admission, we recorded the patient white blood cell count, erythrocyte sedimentation rate, and blood levels of C-reactive protein, procalcitonin, aspartate aminotransferase, alanine aminotransferase, total bilirubin, creatine kinase (CK), CK-MB isoform, brain natriuretic peptide, albumin, potassium, sodium, calcium, creatinine, blood urea nitrogen, fibrinogen (FIB), and D-dimer. We also performed a blood gas analysis and recorded the oxygenation index when the patients were admitted to the intensive care unit (ICU). All patients underwent chest computed tomography examination prior to admission to determine the distribution, number, and types of lung lesions.

### 2.5 Statistical methods

All data were analyzed using SPSS version 25.0 (IBM Corp., Armonk, NY, USA). Categorical variables were reported as frequencies and percentages. The one-sample Kolmogorov-Smirnov test was used to assess whether continuous variables followed a normal distribution. Continuous variables were reported as means and standard deviations or medians and interquartile ranges, depending on whether they had a normal or skewed distribution.

## 3 Results

### 3.1 Patient characteristics

Of the 14 patients, 12 were male (85.71%) and two were female (14.29%), with an average age of 57.21 ± 7.04 years and body mass index of 23.90 ± 1.19 kg/m^2^. Eleven patients had a clear history of poultry contact (92.58%), seven had underlying chronic diseases (50%), and eight had a history of smoking (57.14%) (Table 1).

**Table 1:** Demographic and clinical characteristics of the 14 patients.

Case#	Age (years) and sex	History of smoking	History of avian or poultry contact	Underlying diseases	Symptoms
1	55 M	Yes	Vegetable market vendor	None	Fever, chest tightness, weakness, abdominal distention, vertigo, nausea, vomiting
2	64 M	Yes	Long-term poultry raising	None	Fever, vertigo, weakness, nasal congestion, rhinitis, cough, expectoration, nausea, vomiting
3	58 M	No	Farm worker	Renal transplantation, renal insufficiency, hypertension	Fever, dyspnea, diarrhea, vomiting
4	50 M	No	Unknown	Gouty arthritis	Fever, cough, expectoration, dyspnea
5	59 M	Yes	Poultry butcher	Coronary heart disease, hypertension	Fever, weakness, cough, drowsiness, incoherent speech, mental deterioration
6	46 M	Yes	Kept parrots at home	Hemorrhoids, kidney stones, chronic gastritis	Fever, weakness, dyspnea, cough, expectoration, diarrhea
7	56 M	Yes	Long-term poultry raising	None	Fever, weakness, cough, expectoration
8	62 M	Yes	Frequent visits to the market to buy vegetables	COPD, hypertension, ankylosing spondylitis	Fever, cough, expectoration, dyspnea
9	55 M	Yes	Poultry butcher	None	Fever, headache
10	70 M	Yes	Unknown	Hypertension, cervical spondylosis, lumbar disc herniation	Fever, myalgia, headache, cough
11	60 F	No	Frequent visits to the market to buy vegetables	Chronic hepatitis B, cirrhosis with hepatic decompensation, chronic gastric ulcer	Fever, weakness, headache, myalgia, chest pain, cough
12	45 M	No	Raising chickens at home	None	Fever, vertigo, headache, nausea
13	56 F	No	Unknown	None	Fever, headache, vertigo, cough, expectoration, chest pain
14	65 M	No	Vegetable market vendor	None	Fever, abdominal distention, diarrhea

COPD = chronic obstructive pulmonary disease.

### 3.2 Clinical manifestations of *Chlamydia psittaci* pneumonia

All patients had fever (100%), with headaches, dizziness, muscle aches, fatigue, sneezing, nasal congestion, runny nose, and other flu-like symptoms occurring in 10 patients (71%), cough and sputum in nine patients (64%), nausea, vomiting, abdominal distension, abdominal pain, diarrhea, and other abdominal symptoms in four patients (29%), dyspnea in five patients (36%), altered mental state in one patient (7%), and chest pain in one patient (7%) (Table 1).

### 3.3 Laboratory findings

Blood tests revealed markedly elevated neutrophil percentages, and blood levels of C-reactive protein (CRP), procalcitonin (PCT), brain natriuretic peptide (BNP), lactate dehydrogenase, and CK. Blood level of aspartate aminotransferase, creatinine, urea, FIB, D-dimer, and troponin were slightly elevated; and the albumin, sodium, and calcium levels were slightly decreased. All patients had a significant decrease in oxygen partial pressure and oxygenation index on admission to the ICU (Table 2).

**Table 2:** Laboratory parameters of the fourteen patients.

Parameter (units)	Normal range	Median (range) or (mean ± SD)
Blood tests		
WBC (×10^9^/L)	4–10	7.48 ± 3.29
Neutrophil (%)	50–70	87 (7–95)
HGB (g/L)	120–160	123.2 ± 22.9
PLT (×10^9^/L)	100–300	178 (24–590)
CRP (mg/L)	0–8	150 ± 48
PCT (ng/mL)	0–5	1.6 (0.5–48.8)
AST (U/L)	13–40	78 (13–382)
ALT (U/L)	7–50	47 (9–1621)
TBIL (μmol/L)	1.7–21	15.8 (4.5–61.3)
ALB (g/L)	35–50	31.7 ± 4.5
LDH (U/L)	0–247	387 (180–2348)
CK (U/L)	26–174	567 (25–16620)
CK-MB (U/L)	0–24	19 (3–170)
BNP (pg/mL)	0–100	430 (10–9000)
K (mmol/L)	3.5–5.5	3.7 ± 0.35
Na (mmol/L)	135–145	133.8 ± 4.7
Ca (mmol/L)	2.25–2.75	2.08 ± 0.17
Cr (μmol/L)	59–104	120.3 ± 55.6
BUN (mmol/L)	3.2–7.1	8.53 ± 3.08
FIB (g/L)	2–4	6.2 ± 1.7
D-dimer (mg/L)	0–0.5	2.64 ± 1.51
Cardiac function		
LVEF (%)	50–70	57 ± 13
Respiratory function		
PO_2_ (mmHg)	80–100	74.9 ± 26.4
PaO_2_/FiO_2_ (mmHg)	400–500	233 (205–422)

ALB = albumin, ALT = alanine aminotransferase, AST = aspartate aminotransferase, BNP = brain natriuretic peptide, BUN = blood urea nitrogen, Ca = calcium, CK = creatine kinase, CK-MB = creatine kinase MB isoenzyme, Cr = creatinine, CRP = C-reactive protein, FIB = fibrinogen, HGB = hemoglobin, K = potassium, LDH = lactate dehydrogenase, LVEF = left ventricular ejection fraction, Na = sodium, PaO_2_/FiO_2_ = oxygenation index, PCT = procalcitonin, PLT = platelets, PO_2_ = partial pressure of oxygen, TBIL = total bilirubin, WBC = white blood cells.

### 3.4 Microbiology results

**Figure 1:**
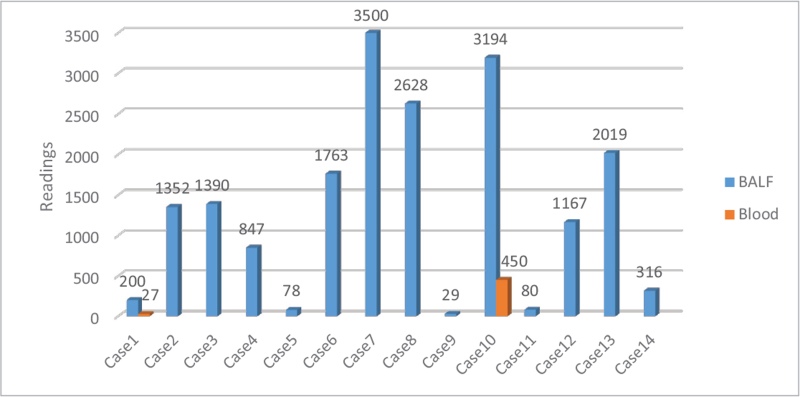
Metagenomic next-generation sequencing results and number of *Chlamydia psittaci* readings obtained for each patient. BALF = bronchoalveolar lavage fluid.

The traditional microbiological examination of the 14 patients, including sputum, blood, and BALF culture, did not reveal any other pathogens. All patients received mNGS testing of the BALF, and two patients had simultaneous BALF and blood testing using mNGS. The median sequence number for mNGS analysis of alveolar lavage fluid samples was 1325.93 ± 1174.14 (Fig. 1). The mNGS sequence numbers in the two blood samples tested were 27 and 450. The sequence numbers for the corresponding BALF samples were 200 and 3194, respectively (Fig. 1). In six patients (42.86%), the mNGS results of alveolar lavage fluid samples also revealed that the numbers of sequences corresponding to *Chlamydia abortus* were 12, 31, 97, 115, 173, and 194.

### 3.5 Chest computed tomography

**Figure 2:**
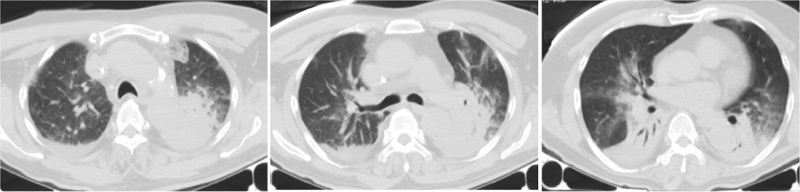
Chest computed tomography of Case 3, a 58-year-old male, showing patchy consolidation of both lungs with blurred edges, and a small right pleural effusion.

**Figure 3:**
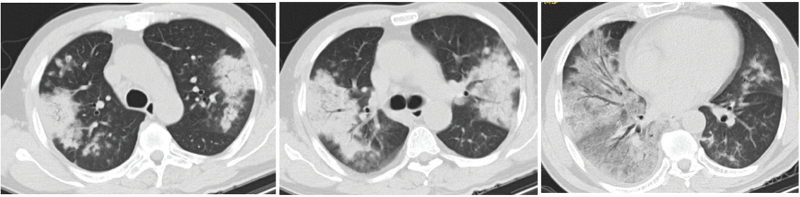
Chest computed tomography of Case 4, a 50-year-old male, showing multiple patchy, patchy and nodular consolidation shadows, ground-glass opacity, air-bronchogram, blurred edges, slightly thickened interlobular septa in both lungs, and a small right pleural effusion.

**Figure 4:**
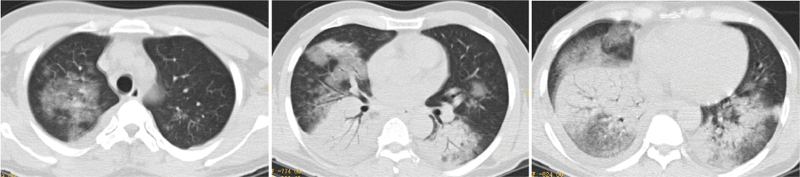
Chest computed tomography of Case 6, a 46-year-old male, showing large consolidation shadows and ground-glass opacity in both lungs, air bronchograms, and a small bilateral pleural effusion.

**Figure 5:**
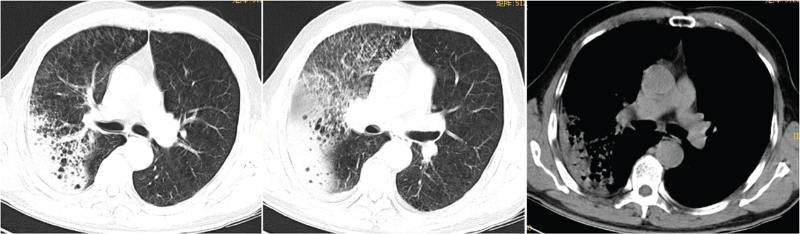
Chest computed tomography of Case 10, a 70-year-old male, showing consolidation shadows in the right lung, scattered cavities, and a small right pleural effusion.

**Figure 6:**
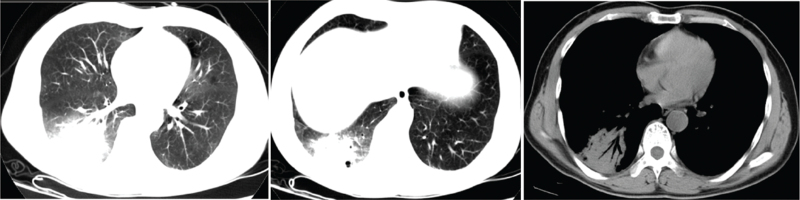
Chest computed tomography of Case 12, a 45-year-old male, showing patchy increased density shadows and consolidation shadows in the right lower lung, with air bronchograms.

All patients underwent preadmission chest computed tomography examination, of which two patients (14%) had left-sided lesions, five patients (36%) had right-sided lesions, seven patients (50%) had bilateral lesions, four patients (29%) had upper lobe lesions, 10 patients (71%) had middle-lower lobe lesions, five patients (36%) had single lobe lesions, and nine patients (64%) had multilobar lesions; reflected as consolidation occurred in six patients (43%), consolidation shadows mixed with ground-glass opacities were present in five patients (36%), two patients (14%) had patchy shadows, and one patient (7%) had consolidation shadows and mixed ground-glass opacities with patchy shadows. Eleven patients (79%) had pleural effusions (Figs. 2–6).

### 3.6 Treatment and outcome

The median time from disease onset to hospital admission for the 14 patients was 4.5 days (interquartile range [IQR]: 1–17 days); the median time from admission to diagnosis was 4.5 days (IQR: 2–12 days); and the mean length of hospital stay was 20.93 ± 8.46 days. Eight patients (57%) were admitted directly to the ICU and six patients (43%) were initially admitted to the general ward and then transferred to the ICU. The median time from hospital admission until transfer to the ICU was 3 days (IQR: 0.5–11 days). The mean Pneumonia Severity Index score of the 14 patients on admission to the ICU was 146.71 ± 21.23, and the median Acute Physiology and Chronic Health Evaluation I score was 17 (IQR: 15–39). The mean time from admission to diagnosis for the 14 patients was 5.1 ± 2.6 days. All 14 patients presented with respiratory failure, and all patients (100%) received invasive mechanical ventilation therapy, of which two patients (14%) received venous-venous extracorporeal membrane oxygenation therapy and four patients received continuous renal replacement therapy. Three of 14 patients (21%) died, and 11 patients (79%) recovered fully and were discharged (Table 3). The three patients who died were all treated with β-amides or β-amides in combination with glycopeptide antibiotics before diagnosis. One patient died of multiple organ failure before the mNGS report was ready, and the other two patients who died were treated with doxycycline in combination with quinolones after being diagnosed. The 11 patients who recovered fully were all treated with β-amides in combination with quinolone antibiotics before the diagnosis and were subsequently treated with either doxycycline alone or doxycycline in combination with a quinolone after diagnosis (Table 3).

**Table 3:** Treatment and outcome of 14 patients with severe *Chlamydia psittaci* pneumonia diagnosed by metagenomic next-generation sequencing.

Case #	Length of hospital stay (days)	Time from admission to diagnosis (days)	PSI score	APACHE II score	Supportive treatment	mNGS *C. psittaci* reads^∗^ (n)	Antibiotics before diagnosis	Antibiotics after diagnosis	Outcome
1	22	5	130	15	Mechanical ventilation, V-V ECMO, CRRT	200 (blood 27)	MEM	DOX + MXF	Death
2	4	3	179	16	Mechanical ventilation	1352	MEM + VAN	NA^†^	Death
3	18	3	146	39	Mechanical ventilation, CRRT	1390	MEM	DOX + MXF	Death
4	34	3	152	17	Mechanical ventilation	847	MEM + MXF	DOX + MXF	Survived
5	15	5	192	24	Mechanical ventilation	78	MEM + MXF	DOX + MXF	Survived
6	18	2	147	16	Mechanical ventilation	1763	MEM + LVX	DOX + LVX	Survived
7	17	4	153	17	Mechanical ventilation	3500	TZP + MXF	DOX + MXF	Survived
8	20	4	139	23	Mechanical ventilation	2628	TZP + LVX	DOX + LVX	Survived
9	18	7	135	15	Mechanical ventilation	29	TZP + LVX	DOX + MXF	Survived
10	40	12	150	30	Mechanical ventilation, V-V ECMO	3194 (blood 450)	TZP + MXF	DOX	Survived
11	29	8	125	18	Mechanical ventilation	80	TZP + LVX	DOX	Survived
12	21	7	165	19	Mechanical ventilation, CRRT	1167	IPM/cilastin + LVX	DOX + LVX	Survived
13	22	3	136	17	Mechanical ventilation, CRRT	2019	TZP + LVX	DOX	Survived
14	15	6	105	17	Mechanical ventilation	316	CFP/SUL +LVX	DOX + LVX	Survived

APACHE = The Acute Physiology and Chronic Health Evaluation, CFP = cefoperazone, CRRT = continuous renal replacement therapy, DOX = doxycycline, IPM = imipenem, LVX = levofloxacin, MEM = meropenem, MXF = moxifloxacin, NA = not applicable, PSI = Pneumonia Severity Index, SUL = sulbactam, TZP = piperacillin-tazobactam, VAN = vancomycin, V-V ECMO = venous-venous extracorporeal membrane oxygenation.

∗ The metagenomic next-generation sequencing was performed on bronchoalveolar lavage fluid unless stated otherwise.

† The patient died before the metagenomic next-generation sequencing results were reported.

## 4 Discussion

*Chlamydia psittaci* can be divided into 10 genotypes, of which genotypes A and E can infect humans.^[5]^ The lungs are the most common site of *C. psittaci* infection. According to Hogerwerf et al,^[6]^*C. psittaci* pneumonia account for approximately 1% of CAP, of which approximately 30% have severe pneumonia.^[6,7]^ One study reported that the carrier rate of *C. psittaci* amongst poultry that were commercially sold in the markets was 13% in chickens, 39% in ducks, and 31% in pigeons.^[8]^ Contact with birds or poultry is regarded as the main risk factor of *C. psittaci* pneumonia,^[9]^ and 11 patients (92.58%) in this study had a history of poultry or bird contact. Hence, newly admitted patients with severe pneumonia should be asked about their poultry contact history. The patients’ average age in this study was 57 years, which was similar to that reported by Rybarczyk et al;^[10]^ male patients accounted for 86%, which was is similar to the male predominance reported by Raeven et al.^[11]^

*Chlamydia psittaci* pneumonia mostly manifests as flu-like symptoms, such as fever, fatigue, myalgia, headache, and cough.^[12]^ In this study, most patients had flu-like symptoms at the early stage. *C. psittaci* infection needs to be ruled out in patients with severe pneumonia and typical flu-like symptoms. In this study, 36% of the patients experienced dyspnea before hospital admission, whereas another study focusing on severe pneumonia caused by *Chlamydia psittaci* reported that 100% of patients had dyspnea. The difference in the prevalence of dyspnea in different studies may be related to differences in the diagnostic classification and treatment approaches between areas. In the study by Chen et al^[13]^ patients were first treated in primary medical institutions and then transferred to higher-level hospitals when their conditions worsened. However, another study focusing on severe pneumonia caused by *Chlamydia psittaci* reported that 100% of patients had dyspnea

In this study, the neutrophil percentage, erythrocyte sedimentation rate, CRP, PCT, lactate dehydrogenase, CK, and BNP were significantly increased. Su et al^[14]^ reported that CK and BNP were independent risk factors for severe *C. psittaci* pneumonia, and PCT was also increased in patients with severe pneumonia. CK is distributed in many tissues and is mainly found in muscle tissues, such as skeletal muscles and cardiac muscle; high levels of CK occur when *C. psittaci* pneumonia is coupled with rhabdomyolysis, of which the mechanism is currently unclear. CK levels greater than 174 U/L comprise a predictive factor for severe *C. psittaci* pneumonia. In addition to being a biomarker for heart failure, BNP is also one of the biomarkers for the severity and prognosis of CAP.^[15]^ The significant elevation of the BNP levels in patients with severe *C. psittaci* pneumonia was probably due to hypoxia and increased pro-inflammatory cytokine secretion.^[16]^ The high levels of inflammatory factors (CRP, PCT) and low oxygenation index observed in this study supports this hypothesis. In this study, the patients’ FIB and D-dimer levels were slightly elevated, as reported by Zhou et al,^[17]^ the severity of CAP is positively correlated with abnormal blood coagulation, which further indicates that *C. psittaci* pneumonia is more likely to develop into severe illness.

It is not common for psittacosis to affect the heart. The CK-MB levels of the patients in this study were not significantly elevated, and the left ventricular ejection fraction did not decrease significantly. The patients’ aspartate aminotransferase, creatinine, and urea levels were elevated, and this occurred in combination with varying degrees of liver and kidney damage, which were complicated by severe renal failure, with four patients receiving continuous renal replacement therapy. Severely ill patients are prone to electrolyte disturbances, such as decreased blood sodium and calcium.

In this study, patients mainly had lesions in the bilateral, middle-lower lobe, and multilobar disease, generally presented as consolidation. In patients with *C. psittaci* pneumonia, the extent of the lesion is closely related to the severity of the disease, and the risk of respiratory failure with multilobar lesions is higher than in other types of CAP. All 14 patients had respiratory failure and received invasive mechanical ventilation, and two of these patients received venous-venous extracorporeal membrane oxygenation therapy Further, 79% of the patients had pleural effusions, which was higher than the incidence rates of 15% reported in Australia^[12]^ and 44.4% reported in another Chinese study.^[13]^ Concomitant pleural effusion can be considered related to the involvement of the pleural membrane and secondary hypoproteinemia.

Metagenomic NGS can be used to rapidly diagnose infectious pathogens, including bacteria, fungi, viruses, mycoplasma, chlamydia, and parasites.^[18]^ For the diagnosis of *C. psittaci* pneumonia, mNGS is not only highly sensitive, but it can also provide pathogen load information (based on the number of reads). In this study, mNGS analysis of the alveolar lavage fluid of six patients concurrently discovered sequences of *C. abortus.* This may be because *C. abortus* and *C. psittaci* have similar genetic backgrounds.^[19]^ In this study, the patients’ time from admission to the diagnosis of *C. psittaci* pneumonia was approximately 5 days. Considering the mNGS costs and timely opportunities for use, it is not accepted as a routine investigation for severe pneumonia. Instead, mNGS testing is recommended only when empirical antibiotic therapy fails.

*Chlamydia psittaci* belongs to the *Chlamydia* genus, and hence, tetracyclines, macrolides, and quinolones can interfere with the synthesis of DNA and protein, which can be used as treatment options.^[20]^ At present, tetracyclines such as doxycycline, tetracycline, oxytetracycline, and minocycline are the first-choice for the treatment of *C. psittaci* pneumonia.^[5]^ Azithromycin and quinolones also have antibacterial activity against *C. psittaci*, of which moxifloxacin works best,^[21]^ whereas azithromycin is considered the best drug alternative for patients with contraindications to tetracyclines and quinolones. However, macrolides might not be effective for patients with severe illness and those who are pregnant.^[2]^ For severely ill patients, oxytetracycline is recommended.^[3]^ Because oxytetracycline is not available in our hospital, we used doxycycline in combination with quinolones to achieve better results. The recommended duration of antibiotic treatment for *C. psittaci* pneumonia is 14 to 21 days to prevent recurrence.^[5]^ The mortality rate of patients with severe *C. psittaci* pneumonia was 21% in this study, which is similar to the 10% to 20% mortality reported by Rybarczyk et al.^[10]^ None of the three patients who died had been treated with antibiotic regimens that included quinolones before diagnosis, and the 11 patients who survived had received β-amides in combination with quinolones before diagnosis. We recommend the empirical use of β-amides in combination with quinolones as empirical antibiotics in critically ill patients before diagnosis.^[3]^ This enables the treatment to be customized after the patient is diagnosed. This empirical antibiotic strategy needs to be validated by prospective studies with a larger sample size.

## 5 Conclusions

In conclusion, poultry contact history and typical flu-like symptoms are early indicators of *C. psittaci* pneumonia, whereas significant increases in PCT, CK, and BNP indicate severe illness. mNGS provides a rapid diagnostic tool. Moreover, anti-infection treatment plans that include quinolones should be used early and empirically for critically ill patients, to gain valuable time for the diagnosis and “targeted” treatment of patients.

## Acknowledgments

We thank the patients enrolled in our study.

## Author contributions

All authors made a significant contribution to the work reported, whether that is in the conception, study design, execution, acquisition of data, analysis and interpretation, or in all these areas; took part in drafting, revising or critically reviewing the article; gave final approval of the version to be published; have agreed on the journal to which the article has been submitted; and agree to be accountable for all aspects of the work. All authors have read and approved the manuscript.

**Conceptualization:** Anbing Zhang, Xiaoling Yuan, Xiuqiong Xia

**Data curation:** Anbing Zhang, Dandan Zhang

**Formal analysis:** Anbing Zhang, Dandan Zhang, Xiuqiong Xia

**Investigation:** Anbing Zhang, Dandan Zhang, Haiming Niu, Xiuqiong Xia, Yanhua Lv, Yuxia Liu

**Methodology:** Anbing Zhang, Dandan Zhang, Haiming Niu, Jianping Liang, Xiaoling Yuan, Xiuqiong Xia, Yuxia Liu

**Project administration:** Dandan Zhang, Jianping Liang, Xiaoling Yuan, Xiuqiong Xia, Yanhua Lv

**Resources:** Anbing Zhang, Jianping Liang, Xiaoling Yuan, Xiuqiong Xia, Yuxia Liu

**Software:** Anbing Zhang, Haiming Niu, Yanhua Lv

**Supervision:** Anbing Zhang, Haiming Niu, Jianping Liang

**Validation:** Anbing Zhang, Jianping Liang, Xiaoling Yuan, Yanhua Lv

**Visualization:** Anbing Zhang, Haiming Niu, Jianping Liang, Xiaoling Yuan, Yanhua Lv

**Writing – original draft:** Anbing Zhang

**Writing – review & editing:** Anbing Zhang, Jianping Liang
